# Draft Genome Sequence of *Salinibacillus aidingensis* Strain MSP4, an Obligate Halophilic Bacterium Isolated from a Salt Crystallizer of the Rann of Kutch, India

**DOI:** 10.1128/genomeA.00253-13

**Published:** 2013-07-05

**Authors:** Kamal Krishna Pal, Rinku Dey, Dharmesh Sherathia, Trupti Dalsania, Kinjal Savsani, Ilaxi Patel, Manesh Thomas, Sucheta Ghorai, Sejal Vanpariya, Rupal Rupapara, Namrata Acharya, Priya Rawal, Pragnesh Joshi, Bhoomika Sukhadiya, Mona Mandaliya, Anil Kumar Saxena

**Affiliations:** Microbiology Section, Directorate of Groundnut Research, Junagadh, Gujarat, Indiaa; Division of Microbiology, Indian Agricultural Research Institute, New Delhi, Indiab

## Abstract

We report the 7.42-Mbp draft whole genome sequence of *Salinibacillus aidingensis* strain MSP4, an obligate halophilic bacterium, isolated from a salt crystallizer of the Rann of Kutch in India. Analysis of the genome of this organism will lead to a better understanding of the genes and metabolic pathways involved in imparting osmotolerance.

## GENOME ANNOUNCEMENT

*Salinibacillus aidingensis* strain MSP4 (16S rRNA GenBank accession number JX518262), an obligate halophilic and endospore-forming bacterium, was isolated from a salt crystallizer of the Rann of Kutch, India. It grows optimally at a 10% (range, 8 to 23%) NaCl concentration in medium at a 37°C temperature and 7.0 pH. The genome of *Salinibacillus aidingensis* MSP4 was sequenced with a view to understanding the mechanism(s) of osmotolerance and to mine relevant gene(s).

The whole genome of MSP4 was sequenced using the Roche 454 Genome Sequencer (GS FLX). Both shotgun and mate-paired library sequencing were performed at Macrogen Inc., South Korea, through Sequencher Tech Pvt. Ltd., Ahmedabad, India. In shotgun sequencing, 761,949 reads of 343,334,611 bases, with an average read length of 450 bp, were generated. In mate-pair libraries, however, 137,026 and 128,147 reads were produced, respectively, with average read lengths of 443 bp and 452 bp, respectively.

The reads were assembled using GS De Novo Assembler v2.6 (1). The genome assembly of *Salinibacillus aidingensis* MSP4 (G+C content of 42.35%) has approximately 61-fold coverage and contains 21 scaffolds of 7,421,686 bp with an average length of 353,413 bp. The scaffolds consist of 77 contigs of 7,387,864 bp with an average length of 95,946 bp. *N*_50_ scaffold lengths of 1,071,925 bp, with the smallest scaffold of 2,944 bp and the largest scaffold of 2,052,512 bp, were obtained. Similarly, for contigs, *N*_50_ contigs with a length of 186,768 bp, with the smallest scaffold contigs of 928 bp and the largest scaffold contigs of 521,343 bp, were obtained. All assembly data were deposited in the DDBJ/EMBL/GenBank nucleotide sequence database.

The draft genome was annotated by the RAST (Rapid Annotation using Subsystem Technology) server (2), Glimmer 3 (3, 4), GeneMark (5, 6), and KEGG database (7). In addition, tRNAScan-SE (8), RNAmmer (9), and Signal P4.1 (10) were used, respectively, for predicting the tRNA and rRNA genes and signal peptides.

Using the different software tools, we predicted 7,667 coding sequences (CDS), with 6,424,323 bp in the CDS. There were 135 RNA-encoding genes (124 tRNA and 11 rRNA genes) and 490 subsystems. Among the CDS, 4,207 are not in the subsystem (nonhypothetical CDS, 1,663; hypothetical CDS, 2,544), whereas 3,460 CDS (nonhypothetical, 3,271; hypothetical, 189) are in the subsystem. In this organism, RAST annotation also revealed the association of 201 genes involved in stress responses (osmotic stress, 37; oxidative stress, 81; cold shock, 4; heat shock, 30; detoxification, 2; periplasmic stress, 1; no subcategory, 46). Use of the Signal P4.1 server predicted 332 signal peptides. A total of 3,516 open reading frames (ORFs) were mapped to different biochemical pathways of KEGG (K00003 to K16706).

Exploring the genome of *Salinibacillus aidingensis* MSP4 will further pave the way for understanding the mechanisms of salinity tolerance and the genes, biochemical pathways, and metabolites involved in osmotolerance. The comparative genome analysis of a number of organisms obtained from the salt crystallizers of the Rann of Kutch of Gujarat, India, and having different levels of osmotolerance is under way.

### Nucleotide sequence accession numbers.

This Whole Genome Shotgun project has been deposited at DDBJ/EMBL/GenBank under the accession number APIS00000000. The version described in this paper is version APIS01000000.

## References

[1] 454 Life Sciences Corporation 2011 454 sequencing system software manual, version 2.6, p 1–288 454 Life Sciences Corporation, Branford, CT

[2] Aziz RK Bartels D Best AA DeJongh M Disz T Edwards RA Formsma K Gerdes S Glass EM Kubal M Meyer F Olsen GJ Olson R Osterman AL Overbeek RA McNeil LK Paarmann D Paczian T Parrello B Pusch GD Reich C Stevens R Vassieva O Vonstein V Wilke A Zagnitko O 2008 The RAST server: rapid annotations using subsystems technology. BMC Genomics 9:75 18261238 10.1186/1471-2164-9-75 PMC2265698

[3] Delcher AL Bratke KA Powers EC Salzberg SL 2007 Identifying bacterial genes and endosymbiont DNA with glimmer. Bioinformatics 23:673–679 17237039 10.1093/bioinformatics/btm009 PMC2387122

[4] Salzberg SL Delcher AL Kasif S White O 1998 Microbial gene identification using interpolated Markov models. Nucleic Acids Res. 26:544–548 9421513 10.1093/nar/26.2.544 PMC147303

[5] Besemer J Lomsadze A Borodovsky M 2001 GeneMarkS: a self-training method for prediction of gene starts in microbial genome. Implications for finding sequence motifs in regulatory regions. Nucleic Acids Res. 29:2607–2618 11410670 10.1093/nar/29.12.2607 PMC55746

[6] Lukashin AV Borodovsky M 1998 GeneMark.hmm: new solutions for gene finding. Nucleic Acids Res. 26:1107–1115 9461475 10.1093/nar/26.4.1107 PMC147337

[7] Kanehisa M Goto S Kawashima S Okuno Y Hattori M 2004 The KEGG resource for deciphering the genome. Nucleic Acids Res. 32:277–280 10.1093/nar/gkh063 PMC308797 14681412

[8] Lowe TM Eddy SR 1997 tRNAscan-SE: a program for improved detection of transfer RNA genes in genomic sequence. Nucleic Acids Res. 25:955–964 9023104 10.1093/nar/25.5.955 PMC146525

[9] Lagesen K Hallin P Rødland EA Staerfeldt HH Rognes T Ussery DW 2007 RNAmmer: consistent and rapid annotation of ribosomal RNA genes. Nucleic Acids Res. 35:3100–3108 17452365 10.1093/nar/gkm160 PMC1888812

[10] Petersen TN Brunak S von Heijne G Nielsen H 2011 SignalP 4.0: discriminating signal peptides from transmembrane regions. Nat. Methods 8:785–786 21959131 10.1038/nmeth.1701

